# Multicystic dysplastic kidneys (MCDK) during prenatal life and postnatal outcome

**DOI:** 10.1007/s00404-025-08197-y

**Published:** 2025-10-17

**Authors:** Charlotte Johanna Marie Reinhardt, Wolfgang Henrich, Stefan Verlohren, Julia Thumfart, Josefine Theresia Koenigbauer

**Affiliations:** 1 Klinik für Geburtsmedizin, Charité - Universitätsmedizin Berlin, Corporate member of Freie Universität Berlin and Humboldt-Universität zu Berlin, Luisenstraße 64, 10117 Berlin, Germany; 2 Klinik für Pädiatrie mit Schwerpunkt Gastroenterologie, Nephrologie und Stoffwechselmedizin, Charité - Universitätsmedizin Berlin, Corporate member of Freie Universität Berlin and Humboldt-Universität zu Berlin, Augustenburger Platz 1, 13353 Berlin, Germany

**Keywords:** MCDK, Cystic kidney disease, CAKUT, Prenatal ultrasound

## Abstract

**Purpose:**

Multicystic dysplastic kidneys (MCDK) are commonly detected on prenatal ultrasound examinations, occurring in 1 in 1,000 to 4,300 live births. This study focuses on neonatal outcomes in fetuses with MCDK, with particular attention to the presence of additional anomalies.

**Methods:**

Retrospective data of fetuses diagnosed with MCDK at Charité University Hospital between 2005 and 2022 were collected and analyzed. Rates of termination of pregnancy (TOP), intrauterine fetal demise (IUFD) and live birth were evaluated. Outcome parameters, including APGAR score, survival, neonatal ventilation therapy or respiratory adjustment disorder, were examined.

**Results:**

A total of 103 fetuses with MCDK were identified. Eight exhibited bilateral MCDK (7.8%). 92.2% showed unilateral MCDK (n = 95), of which 43 revealed additional anomalies (45.3%). Fetuses with additional anomalies displayed significantly fewer live births, more prematurity, lower APGAR scores and lower gestational ages at birth. The expected outcome of fetuses with MCDK is contingent upon the presence of additional anomalies. Bilateral MCDK, as well as unilateral MCDK with additional anomalies, are associated with an unfavorable postnatal outcome.

**Conclusion:**

To consult parents on the potential postnatal well-being of their offspring with MCDK, it is crucial to search for additional anomalies. Genetic counseling and invasive genetic testing should be offered.

## Introduction

Congenital malformations of the kidney and urinary tract (CAKUT) constitute a heterogeneous group of fetal malformations that affect the development as well as the function of the kidneys and their outflow tracts. The prevalence of CAKUT is 4–60 in 10,000 live births, and it is responsible for about 40–50% of all end-stage kidney diseases (ESKD) in children and adolescents and for about 7% in adulthood [1, 2]. CAKUT can have a genetic or non-genetic background. Many genes associated with CAKUT have been identified, and a genetic cause is described in 9% to 25% with increasing prevalence due to better genetic diagnostics [1, 3–6]. Teratogens can lead to impaired renal development as well. ACE inhibitors, angiotensin receptor blockers, NSAIDs, corticosteroids, immunosuppressants and certain antibiotics are described to impact renal development and function and should be avoided during pregnancy [7]. There is a higher risk for developing CAKUT in the presence of maternal diabetes [3, 8].

Multicystic dysplastic kidneys (MCDK) represent the second most prevalent genitourinary anomaly after congenital hydronephrosis including ureter obstruction with a prevalence of one in 1,000 to one in 4,300 live births [2, 9, 10]. Bilateral MCDK is described in one in 10,000 live births [11]. A slight male predominance has been observed in the occurrence of MCDK in several studies [12–15]. In seven to 14% of MCDK cases, chromosomal abnormalities, copy number variations (CNVs) and syndromes can be identified [10, 11, 16, 17]. However, the pathogenesis of MCDK is not fully understood yet. Embryologically, MCDK as a dysplastic disease is thought to originate from an impaired fusion of the ureteric bud with the renal mesenchyme [14]. Another potential cause of MCDK is ischemia of the metanephros and ureteric bud [12, 18].

MCDK is associated with a near total to total loss of renal function of the affected kidney [15]. However, unilateral MCDK with normal contralateral renal function is associated with a favorable postnatal prognosis [10, 19]. Consequently, bilateral MCDK results in a critical loss of renal function, leading to a significant reduction of amniotic fluid and subsequent pulmonary hypoplasia with an unfavorable prognosis [11, 19, 20].

It has been suggested that an adverse fetal outcome might be associated with additional extra-renal findings detected prenatally [21]. This retrospective analysis investigates whether fetuses with MCDK can be categorized into different groups regarding the expected outcome based on the presence of additional anomalies. Furthermore, this study analyzes possible predictive ultrasound parameters that may forecast the perinatal and long-term prognosis of fetuses with MCDK. The aim was to identify potential predictors for neonatal outcome that might facilitate counseling.

### Prenatal ultrasound

**Fig. 1:**
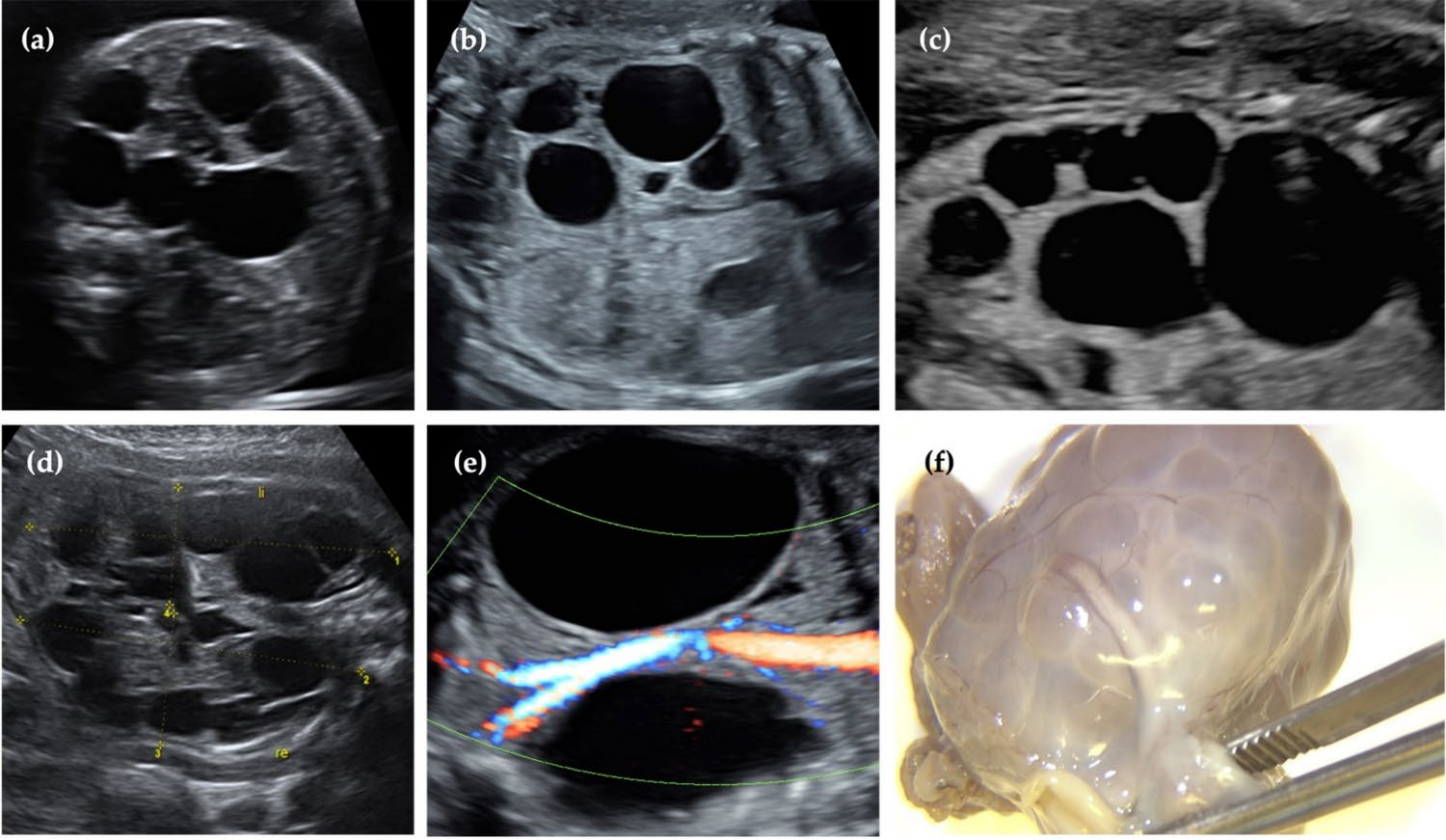
Prenatal diagnosis of multicystic dysplastic kidneys (MCDK): **a** fetal abdomen in 26 + 0 weeks of gestation showing unilateral MCDK, with the enlarged kidney crossing the median sagittal plane, the contralateral kidney appearing normal; **b** fetal abdomen in 20 + 2 weeks of gestation with unilateral MCDK; **c** fetal abdomen in 29 + 6 weeks of gestation displaying unilateral MCDK; **d** fetal abdomen in 29 + 1 weeks of gestation showing bilateral MCDK, both kidneys enlarged, with no normal remaining renal parenchyma; **e** fetal abdomen with bilateral MCDK with large cysts in 26 + 1 weeks of gestation; **f** postmortem macroscopic image of MCDK following TOP

Second trimester anomaly scans have been demonstrated to have high sensitivity for the identification of cystic kidney anomalies during fetal life [1, 10, 22]. The typical ultrasound image of MCDK is characterized by the presence of multiple, non-communicating renal cysts without normal renal structure or regular renal parenchyma [19, 23]. Affected kidneys may be enlarged or even reach beyond the median sagittal plane [19]. The cysts appear to have thin walls, creating an uneven outer renal contour. The remaining parenchyma between the cysts may appear hyperechogenic, and no renal pelvis can be identified [14, 24]. For typical ultrasonographic and macroscopic findings of MCDK, see Fig. 1 (a-f).

### Genetic evaluation and differential diagnoses cystic kidney diseases

MCDK is a congenital renal anomaly often detected via prenatal ultrasound. While typically sporadic, MCDK can be associated with underlying genetic variants or syndromic conditions. Cystic kidney malformations and their potentially underlying genetic causes are associated with a wide spectrum of postnatal outcomes [25]. Therefore, genetic counseling and diagnostic procedures including chromosome analysis, chromosomal microarray analysis (CMA) and exome sequencing should be considered and can be offered to affected families [9, 19]. Genetic testing can aid identifying genes associated with congenital anomalies of the kidney and urinary tract (CAKUT) and MCDK [3].

In 7–15% of patients with kidney cystic disease, a genetic cause has been reported [9, 10, 17, 19]. Several genetic syndromes, such as Zellweger syndrome, VACTERL association, Eagle-Barrett syndrome, renal–hepatic–pancreatic dysplasia (RHPD), Branchio-oto-renal (BOR) syndrome, HNF1B nephropathy and renal coloboma syndrome (RCS), have been linked to kidney cystic disease [9, 18, 23, 26, 27]. Distinguishing the different forms of kidney cystic disease is critical for appropriate pre- and postnatal management [27].

Unilateral MCDK, particularly in the absence of additional anomalies, is predominantly sporadic, and a genetic cause is considered rare [9, 25, 28]. However, the introduction of CMA has identified several CNVs associated with isolated, unilateral MCDK [17]. In contrast, bilateral MCDK and occurrence with additional anomalies are more frequently associated with a genetic variant [9].

**Fig. 2:**
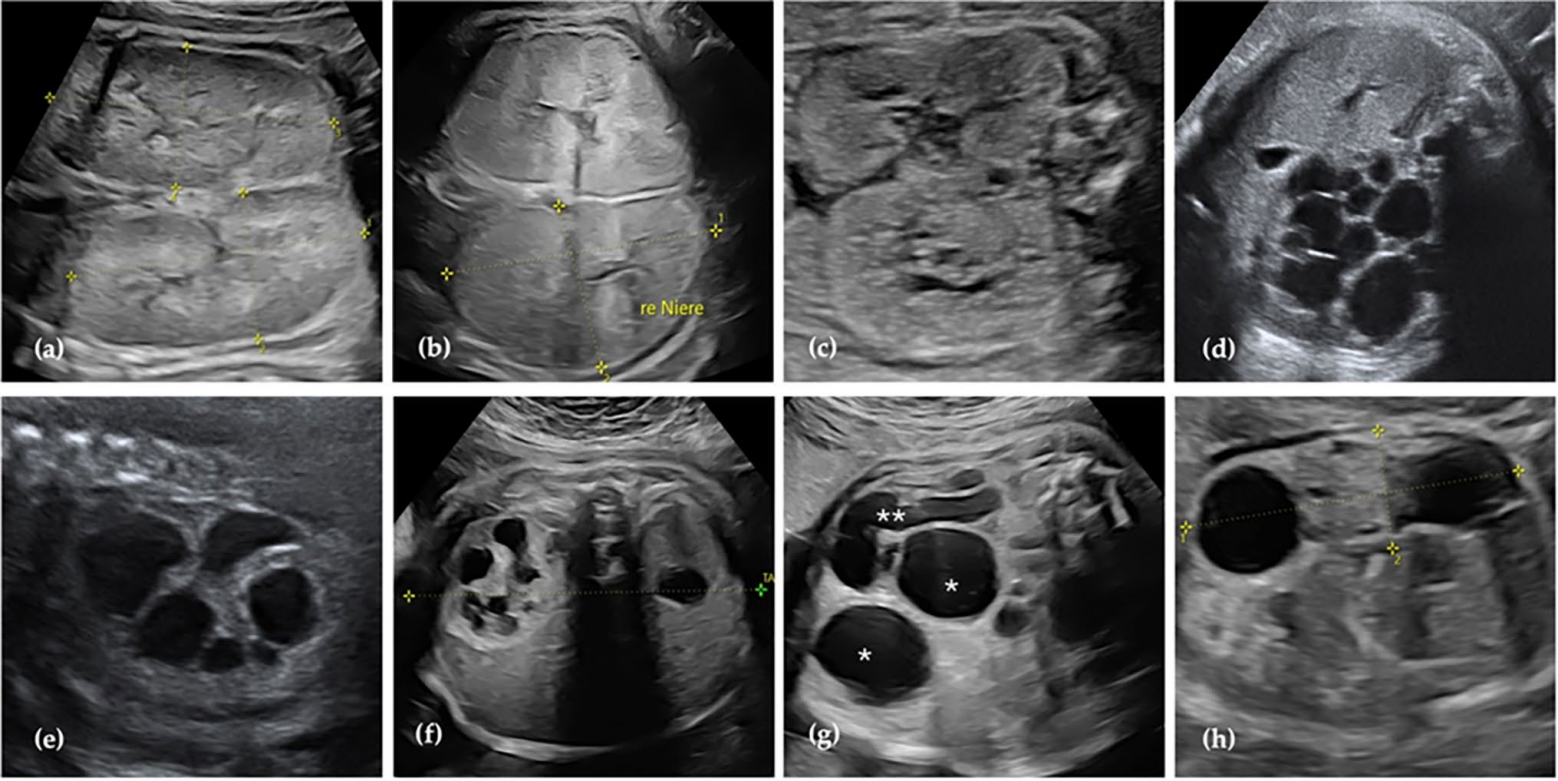
Appearance of cystic kidneys in different diseases prenatally: **a** ARPKD in 33 + 2 weeks of gestation; **b** HNF1B nephropathy in 26 + 3 weeks of gestation; **c** Bardet–Biedl syndrome in 28 + 2 weeks of gestation; **d** abdominal lymphangioma in 38 + 4 weeks of gestation; **e** hydronephrosis in 30 + 0 weeks of gestation; **f** duplex kidney with hydronephrosis in 31 + 3 weeks of gestation; **g** duplex kidney with VUR (confirmed postnatally), hydronephrosis (*) and megaureter (**) in 32 + 3 weeks of gestation; **h** cystic kidney dysplasia in fetus with complex of anomalies including LUTO, hydronephrosis, megaureters, and megacystis and anal atresia in 33 + 6 weeks of gestation

Due to overlapping sonographic features, MCDK can be misdiagnosed as various other renal pathologies, necessitating meticulous ultrasound evaluation of the kidney morphology (Fig. 2a–h) [20, 25, 27]. Cystic kidney diseases can arise from both syndromic and non-syndromic conditions caused by single or multiple gene variants. Given the phenotypic similarity between different cystic kidney diseases, genetic testing is often required to establish an accurate diagnosis [29]. Additionally, lower urinary tract obstruction (LUTO) can lead to cystic renal dysplasia, making it essential to exclude obstructive uropathy when evaluating kidney cysts [30]. Polycystic kidney diseases are inherited ciliopathies that can be clearly distinguished from MCDK in ultrasound due to their common appearance with bilateral cysts and hyperechogenic, bilaterally enlarged kidneys [9, 25, 29]. Autosomal recessive polycystic kidney disease (ARPKD) more commonly appears with abnormal ultrasound parameters prenatally than autosomal dominant polycystic kidney disease (ADPKD) [29].

Other conditions that may resemble MCDK on prenatal ultrasound include hydronephrosis, duplex kidneys and rare entities such as cystic renal tumors and lymphangioma [10]. Figure 2 illustrates different diseases with cystic kidneys observed in prenatal ultrasound.

## Material and methods

In this retrospective study, the prenatal ultrasound database of the Charité University Hospital (Campus Charité Mitte and Charité Campus Virchow-Klinikum) a tertiary perinatal center, prenatal diagnosis and fetal therapy expert clinic in Berlin, Germany, was searched from January 1st 2005 December 31st 2022 using the software ‘ViewPoint™ 5’ and ‘ViewPoint™ 6’ (GE HealthCare, Chicago, USA) for the following terms: MCDK, MCKD, multicystic, kidney cyst, cystic kidney. Because the examinations found were carried out by several certified ultrasound specialists, different terms were used top and therefore needed to be used as search terms. The study was approved by the Charité University Hospital ethics committee on September 11th, 2023 (EA2/158/23).

A total number of 116 fetuses with the prenatal diagnosis of MCDK were identified and included into the analysis. All were diagnosed by a specialist for prenatal ultrasound. Cases were screened and included into the analysis by a physician with advanced prenatal ultrasound knowledge (CR) and confirmed by a certified expert for prenatal ultrasound (JK, certified by the German Society of Ultrasound in Medicine = DEGUM). All fetuses received a structured fetal anomaly scan by an expert for prenatal ultrasound to evaluate structural anomalies. Ultrasonographic examinations were carried out transabdominally using GE Voluson E10, E8 and E6 ultrasound device with curved array transducers with a bandwidth of two to five MHz. Only fetuses with the definite and during data collection again by reassessing the deposited ultrasound images verified diagnosis MCDK were included; those with a different diagnosis were excluded. In the advent of a live birth, postnatal data were assessed from hospital files, cross-checked and correlated with the prenatal findings. Postnatal reassessment of the diagnosis MCDK by ultrasound or scintigraphy was rarely available. The following data were collected for each case, if available: maternal/gestational history including gravida, para, BMI during pregnancy, family history, gestational week when first diagnosed, maternal or paternal kidneys affected. Fetal findings including gender, laterality, pelvic kidney, double kidney, enlarged kidneys, hydronephrosis, pyelectasia, enlarged ureter (megaureter), additional abnormalities within and outside the urinary tract, pulmonary hypoplasia, empty urinary bladder, anhydramnios, oligohydramnios, polyhydramnios, single umbilical artery and genetic diagnostics were assessed. The perinatal outcome was analyzed including pregnancy outcome (live birth, palliative birth, intrauterine fetal death (IUFD) or termination of pregnancy (TOP)), gestational week at delivery, birth mode, APGAR score, percentile of fetal weight, pH in umbilical artery immediately after birth, transfer to neonatal intensive care unit, fetal death within seven days postnatally, adaption disorder. If available postnatal follow-up was examined, including postnatal diagnosis, number of hospitalizations during first year of life, electrolyte disturbance, postnatal operations, need of infusion therapy, additional anomalies diagnosed postnatally, vesicoureteral reflux, lower urinary tract obstructions, required neonatal ventilation therapy, postnatal genetic diagnostics, need of renal replacement therapy, nephrectomy, kidney transplantation, maximum creatinine, hemoglobin postnatally, silent kidney, required prophylactic antibiotic therapy, urinary tract infection requiring antibiotics, kidney involution, malignant changes, survival over one, three, five or 10 years.

The fetuses were classified into unilateral MCDK and bilateral MCDK. The rate of TOP, IUFD, live birth and live birth with palliative care were assessed among other parameters. Additionally, if conducted, genetic test results were reviewed. Ultrasound parameters compiled were assessed toward their significance regarding certain outcome parameters. Data analysis was performed using IBM SPSS Statistics, Version 29. To compare proportions between groups, Chi-square test and Mann–Whitney *U* test was performed. A *p* value below 0.05 was considered to be statistically significant.

## Results

A total of 116 fetuses with the prenatal diagnosis of MCDK were identified. 13 fetuses were misdiagnosed prenatally and revealed a different diagnosis postnatally (11.21%) resulting in a diagnostic accuracy of 88.79%. Among the fetuses with postnatally revised diagnosis, the majority were found to have alternative renal pathologies. In our cohort, the final diagnoses of initially suspected MCDK included one child with ectopic kidney, one with VUR, one with ureterocele, one with duplex kidney and VUR, one with duplex kidney and hydronephrosis and one with cystic kidney dysplasia with a complex of anomalies consisting of LUTO, anal atresia, bilateral hydronephrosis, megaureter and megacystis. Furthermore, there was one child with HNF1B nephropathy, one with hydronephrosis, two with cystic kidney dysplasia (one of them with LUTO), one renal agenesis, one ampullary kidney pelvis syndrome and one abdominal lymphangioma. The following analysis and results refer to the 103 cases in which MCDK was the final diagnosis. The mean maternal age at diagnosis was 30.69 years (± 5.71). The mean gestational age at sonographic diagnosis was 23.21 (± 4.34) weeks of pregnancy with a range from 14 to 39 weeks of pregnancy. The mean gestational age at the initial presentation of MCDK on ultrasound was 22.68 (± 4.30). The mean gestational age at which genetic diagnostics were performed was 21.95 (± 3.14). Genetic diagnostics were performed in 31 pregnancies (30.10%) of which 24 (77.42%) were invasive diagnostics (20 amniocenteses, 2 chorionic villous samplings and 2 fetal blood draws) and 7 (22.58%) were non-invasive prenatal testing (NIPT). Two fetuses were identified with a pathological genetic result (one with trisomy 18 and one with POMT1-gene-variant in a family with members affected by muscular dystrophy). Despite professionals offering to perform genetic counseling and genetic testing, 28 women (27.18%) declined.

### Bilateral MCDK

Eight fetuses displayed bilateral MCDK (7.77%) of which four fetuses demonstrated no additional anomalies (50%). Four fetuses with bilateral MCDK were diagnosed with other findings (50%) that were double bubble sign, single umbilical artery, hyperechogenic intestine and cardiac hypertrophy. Five fetuses (62.50%) with bilateral MCDK were male; the gender of the remaining fetuses could not be specified due to extreme preterm birth. Bilateral MCDK was associated with an adverse perinatal outcome. Four pregnancies were terminated (50%). Four live births occurred of which three received a palliative postnatal care (75%), which resulted in neonatal death within hours after birth. One neonate deceased six days after birth despite maximum therapy. The mean fetal weight percentile at birth or the last percentile measured on ultrasound was 32.71 (± 33.31; median 20.00).

### Unilateral MCDK

**Fig. 3:**
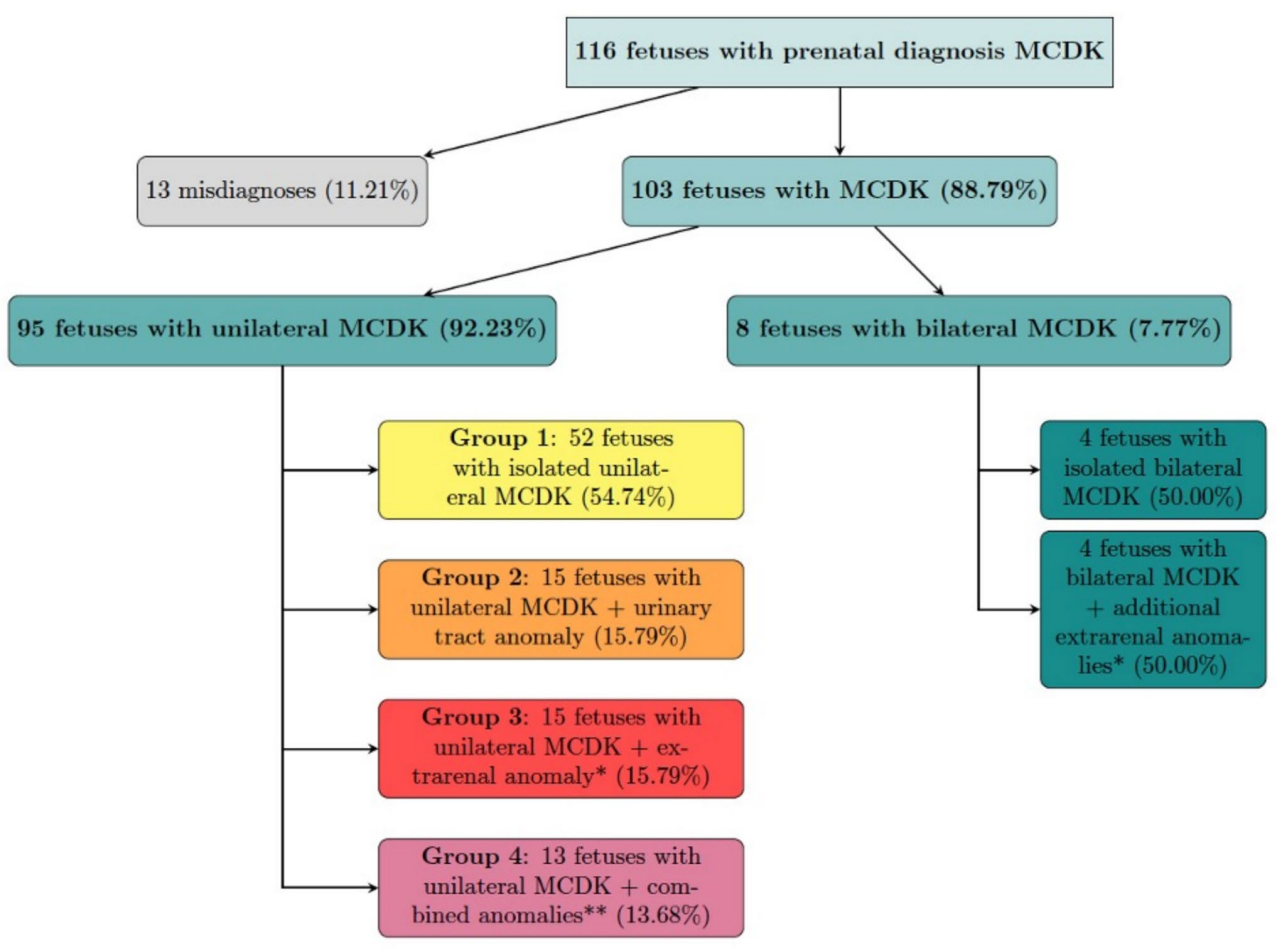
Prenatal MCDK cases are distributed into confirmed MCDK (unilateral and bilateral MCDK) as well as misdiagnosis. Furthermore, fetuses with uni- and bilateral MCDK were divided into groups regarding associated anomalies. **Group 1**: Fetuses with unilateral MCDK without further anomalies, **group 2**: Fetuses with unilateral MCDK plus urinary tract anomalies, **group 3**: Fetuses with unilateral MCDK plus extra-renal anomalies*, **group 4**: Fetuses with unilateral MCDK plus combined anomalies**. *: extra-renal anomalies excluding the urinary tract. **: anomaly of the urinary tract AND extra-renal anomaly apart from the urinary tract

95 fetuses were diagnosed with unilateral MCDK (92,23%). Overall, 51 fetuses displayed a right sided MCDK (53.68%). 43 fetuses were female (45.26%); 52 fetuses were male (54,74%). 52 fetuses had an isolated unilateral MCDK without further malformation (54.74%) (= **group 1**). 15 fetuses with unilateral MCDK revealed additional abnormalities of the urinary tract (15.7%) (= **group 2**); extra-renal findings excluding the urinary tract were detected in 15 fetuses (15.79%) (= **group 3**). 13 fetuses were identified with both extra-renal anomalies and malformations of the urinary system (13.68%) (= **group 4**). Figure 3 (group 1-group 4) shows the distribution of cases included in this study. Extra-renal abnormalities apart from the urinary tract, that were noted in this study, were cardiac anomalies (*n* = 10), single umbilical artery (*n* = 10) and central nervous system malformations (*n* = 10). Anomalies of the urinary tract were contralateral hydronephrosis (*n* = 5), pelvic kidney/ectopic renal tissue (*n* = 8), contralateral pyelectasis (*n* = 4), duplex kidney (*n* = 1), contralateral megaureter (*n* = 1) and ipsilateral megaureter (*n* = 1). Enlarged contralateral kidney in the presence of unilateral MCDK was considered as a compensatory enlargement. It was detected in 20 fetuses (21.05%). The mean fetal weight percentile at birth or the last percentile measured on ultrasound was 45.25 (± 31.32; median 41.50). The weight distribution among the cohort was non-normal. Postnatal data were available for 81 infants out of 83 live births with unilateral MCDK (97.59%).

### Pregnancy outcome

**Fig. 4:**
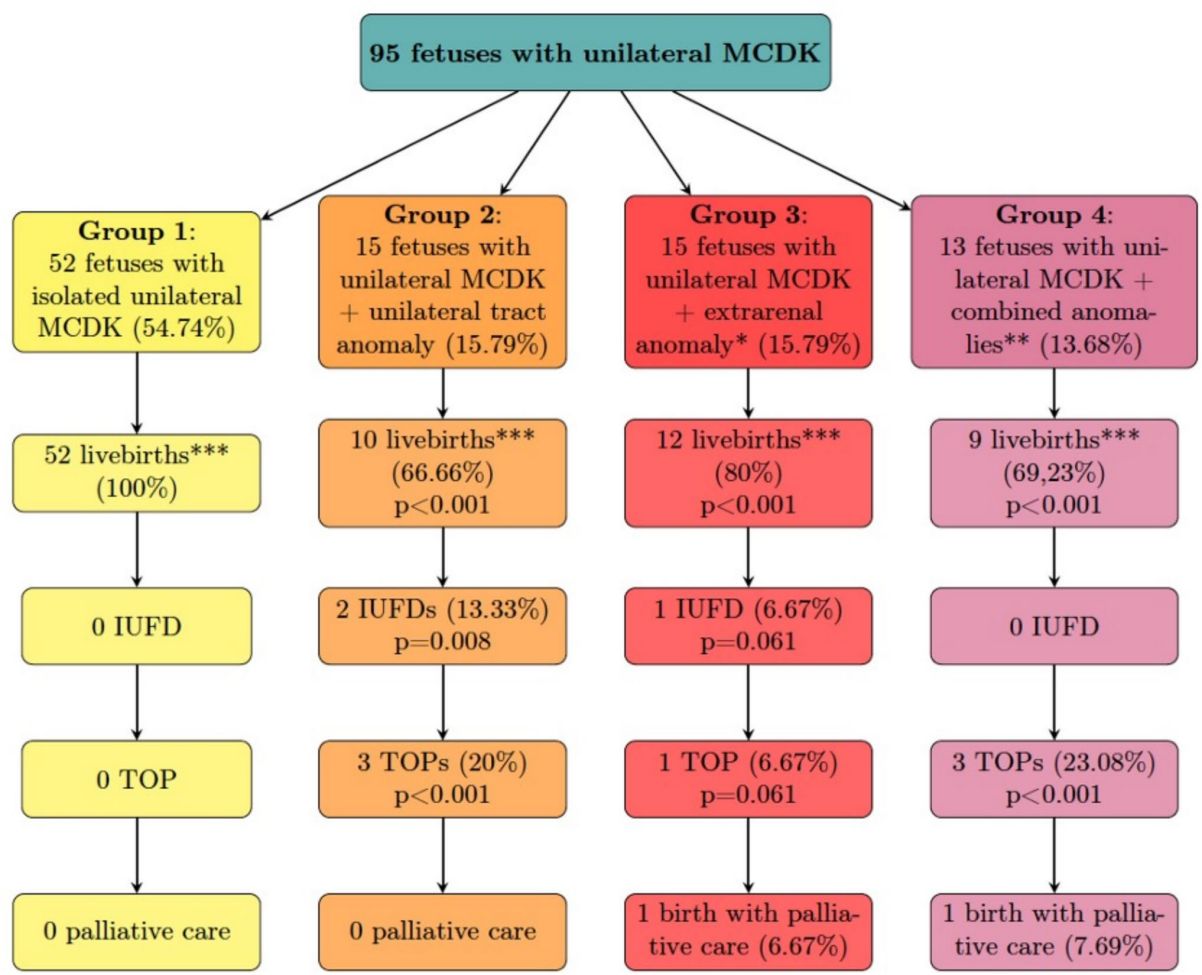
Pregnancy outcome in case of unilateral MCDK divided into groups: **group 1**: Fetuses with unilateral MCDK without further anomalies, **group 2**: Fetuses with unilateral MCDK plus urinary tract anomalies, **group 3**: Fetuses with unilateral MCDK plus extra-renal anomalies*, **group 4**: Fetuses with unilateral MCDK plus combined anomalies**. *: extra-renal anomalies excluding the urinary tract. **: anomaly of the urinary tract AND extra-renal anomaly apart from the urinary tract. ***: excluding births with palliative care

Pregnancy outcomes were analyzed using Chi-square test. The analysis focused on whether the fetus was born alive (live birth), deceased intrauterine (IUFD), if termination of pregnancy was performed due to unfavorable fetal prognosis (TOP), if palliative care was offered postnatally, whether the child was born prematurely (before 37 + 0 weeks of gestation) and the rate of fetal growth restriction (FGR). Furthermore, APGAR score results, fetal pH in the umbilical artery postnatally and the gestational age at delivery were compared using Mann–Whitney U test (Fig. 4 (group 1-group 4) and Table 1). Pregnancy outcomes defined by whether the pregnancy resulted in live birth excluding births with palliative care, TOP, IUFD or palliative birth were analyzed with Chi-square test. Outcomes of group 2, group 3 and group 4 were compared to outcomes of group 1 (Fig. 4, Table 1). Fetuses from group 1 were born alive in 100%, compared to group 2 in 66.66% (*p* < 0.001), group 3 in 80% (*p* < 0.001 compared to isolated MCDK), group 4 in 69.23% (*p* < 0.001 compared to isolated MCDK). There was no IUFD within group 1 and 4, but two fetuses (13.33%) deceased during fetal life in group 2 (*p* = 0.008) and one fetus (6.67%) in group 3 (*p* = 0.061). No pregnancy within group 1 was terminated. TOP was performed in three pregnancies (20.00%) in group 2 (*p* < 0.001), in one within group 3 (6.67%, *p* = 0.061) and in three in group 4 (23.08%, *p* < 0.001). One newborn in group 3 (6.67%, *p* = 0.061) as well as one newborn in group 4 (7.69%, *p* = 0.044) received palliative care postnatally.

**Table 1:** Pregnancy outcome (live birth, IUFD, TOP and palliative care) and fetal outcome regarding maturity at birth, gestational age at birth, fetal growth restriction (FGR), APGAR, mean umbilical cord arterial blood pH and maximum serum creatinine levels in fetuses with MCDK plus anomalies of the urinary tract (group 2), fetuses with MCDK plus extra-renal anomalies* (group 3) and fetuses with MCDK plus combined anomalies** (group 4) compared to fetuses with isolated MCDK (group 1)

Cohort outcome parameter	Group 1 (*n* = 52)	Group 2 (*n* = 15)	*p* value	Group 3 (*n* = 15)	*p* value	Group 4 (*n* = 13)	*p* value
Live birth***	52 (100%)	10 (66.66%)	< 0.001	12 (80%)	< 0.001	9 (69.23%)	< 0.001
IUFD	0	2 (13.33%)	0.008	1 (6.67%)	0.061	0	N/A
TOP	0	3 (20.00%)	< 0.001	1 (6.67%)	0.061	3 (23.08%)	< 0.001
Palliative care	0	0	N/A	1 (6.67%)	0.061	1 (7.69%)	0.044
Premature birth (< 37 + 0 weeks of gestation)	7 (15.55%)	7 (46.66%)	0.005	6 (40%)	0.022	8 (61.54%)	< 0.001
Median gestational age at delivery in weeks of gestation (IQR)	40 (39–41)	39 (26–41)	0.200	38 (34–40)	0.015	33 (28–39,5)	0.002
FGR (fetal weight < 10. Percentile)	1 (0.02%)	1 (0.08%)	0.281	1 (0.067%)	0.342	0	0.614
APGAR1 median (IQR)	9 (9–9)	9 (9–9)	0.904	9 (6.50–9)	0.065	7.5 (2–9)	0.003
APGAR1 < 7	3 (5.8%)	5 (33.3%)	0.435	3 (20%)	0.060	4 (30.8%)	0.002
APGAR5 median (IQR)	10 (9–10)	10 (9.75–10)	0.357	9 (8–10)	0.093	8.5 (1.75–10)	0.005
APGAR5 < 7	1 (1.9%)	5 (33.33%)	0.661	2 (13.3%)	0.617	4 (30.8%)	< 0.001
APGAR10 median (IQR)	10 (10–10)	10 (10–10)	0.381	10 (8–10)	0.063	9 (1–10)	0.005
APGAR10 < 7	1 (1.9%)	5 (33.33%)	0.661	2 (13.3.%)	0.617	4 (30.8%)	< 0.001
Mean umbilical cord arterial blood pH (SD)	7.26 (0.07)	7.26 (0.08)	0.985	7.26 (0.08)	0.837	7.22 (0.09)	0.359
Mean maximum serum creatinine level in mg/dl (IQR)	0.56 (0.28–0.79)	1.21 (0.80–1.71)	0.003	0.82 (0.39–1.22)	0.061	1.40 (0.63–2.5)	0.069

Results in *n* (%) excluding APGAR (median; 25th to 75th percentile), mean pH (median; standard deviation), gestational age at delivery in weeks of gestation (median; 25th to 75th percentile) and mean maximum serum creatinine levels in mg/dl (mean; 25th to 75th percentile; note: not available for all cases due to retrospective design). Chi-square test and Mann–Whitney *U* test were performed

*: extra-renal anomalies excluding the urinary tract

**: anomaly of the urinary tract AND extra-renal anomaly apart from the urinary tract

***: excluding births with palliative care

**Fig. 5:**
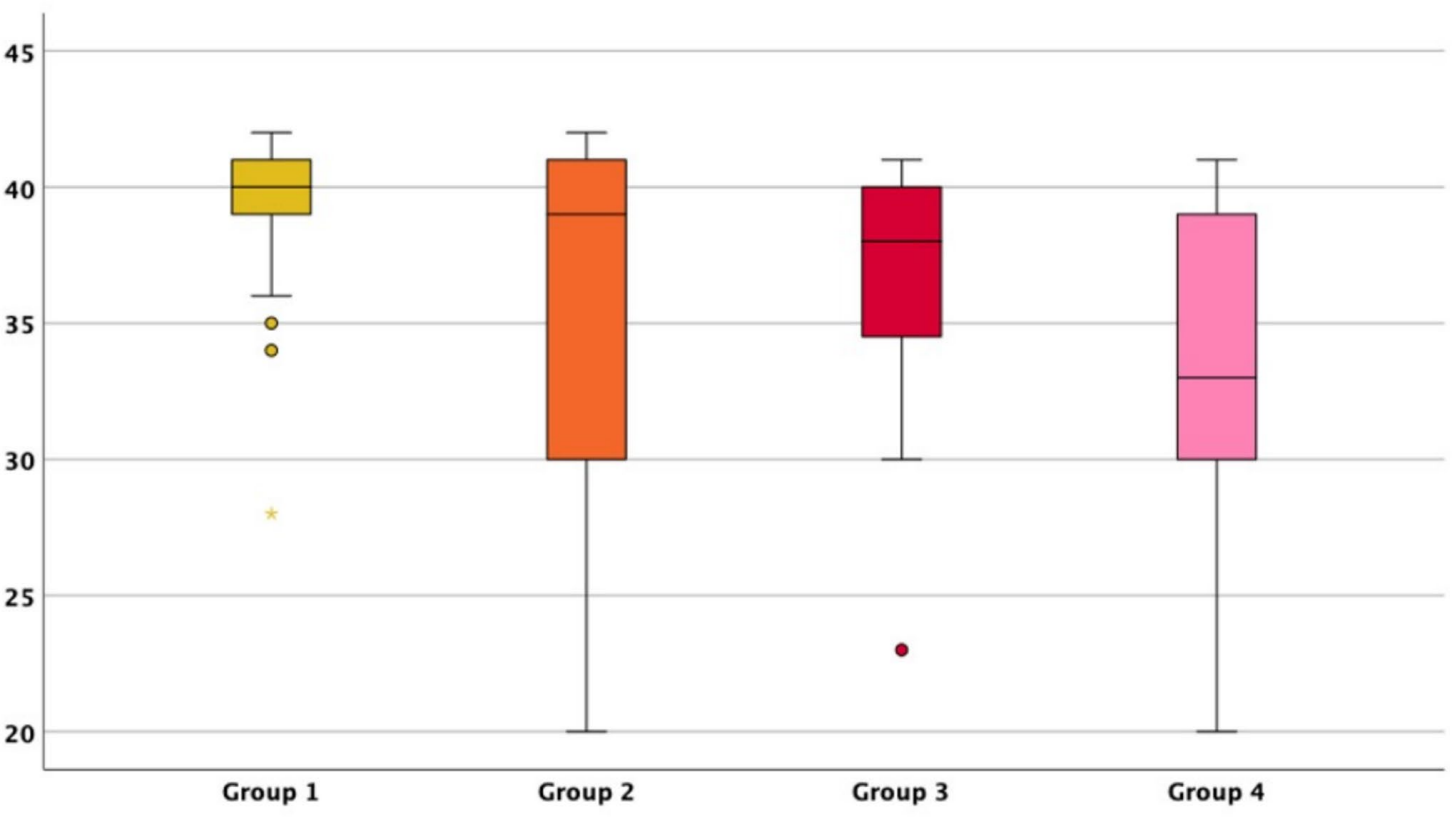
Boxplot illustrating the gestational week at birth (y-axis) of live born fetuses (excluding palliative care) in MCDK-cohorts (x-axis): **isolated unilateral MCDK (group 1)**, **unilateral MCDK plus urinary tract anomalies (group 2)**, unilateral MCDK plus extra-renal anomalies* (group 3) and unilateral MCDK plus combined anomalies** (group 4). The bold horizontal lines show the median, the edges of the boxes indicate 25th and 75th percentiles, whiskers indicate highest and lowest values. Circles indicate mild outliers. *: extra-renal anomalies excluding the urinary tract. **: anomaly of the urinary tract AND extra-renal anomaly apart from the urinary tract

Premature birth was significantly higher in group 2, group 3 and group 4 compared to group 1. The largest percentage of premature deliveries appeared within group 4 (61.54%, *p* < 0.001). The mean gestational age at birth was significantly lower in group 4 with 33.23 gestational weeks (median 33) compared to 39.35 (median 40) gestational weeks in group 1 (*p* = 0.002) (Fig. 5, group 1-group 4).

**Fig. 6:**
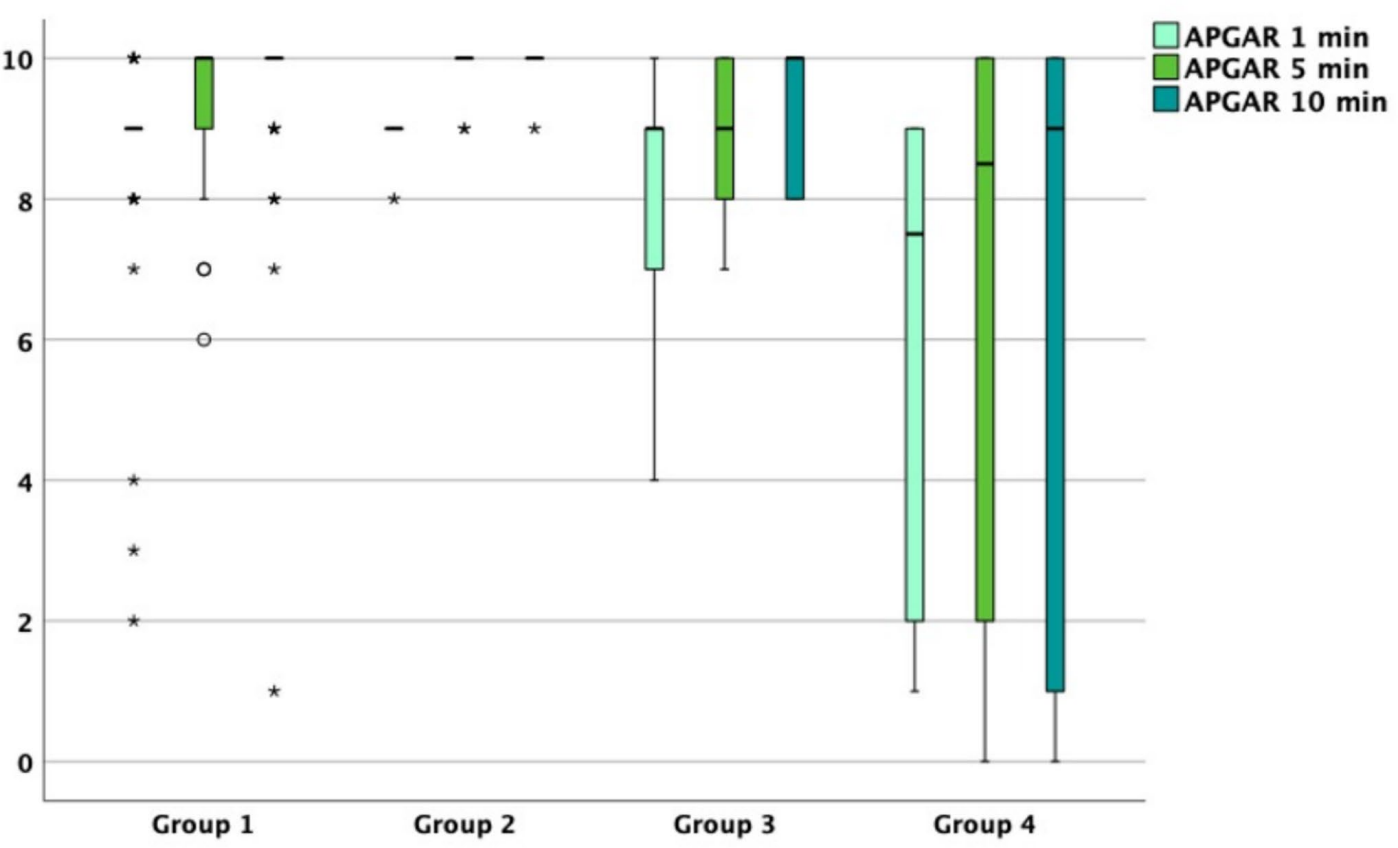
Boxplot illustrating APGAR score results (y-axis) in MCDK-cohorts (x-axis): **isolated unilateral MCDK (group 1)**, **unilateral MCDK plus urinary tract anomalies (group 2)**, unilateral MCDK plus extra-renal anomalies* (group 3) and unilateral MCDK plus combined anomalies** (group 4). The bold horizontal lines show the median, the edges of the boxes indicate 25th and 75th percentiles, whiskers indicate highest and lowest values. Circles mark mild outliers; crosses mark extreme outliers. *: extra-renal anomalies excluding the urinary tract. **: anomaly of the urinary tract AND extra-renal anomaly apart from the urinary tract

No significant differences could be seen between the rates of FGR between the groups. The median APGAR score was significantly lower in neonates from group 4 with 7.5/8.5/9 at 1/5/10 min compared to group 1 with 9/10/10 at 1/5/10 min of life (*p* = 0.003/0.005/0.005) (Fig. 6, group 1-group 4)).

The mean arterial pH did not reveal any significant differences. The mean maximum serum creatinine levels documented postnatally were the highest among group 4 (1.40) but differed significantly between group 1 (0.56) and group 2 (0.21, *p* = 0.003).

### Short- and long-term fetal outcome

Short- and long-term neonatal outcome was analyzed and compared between groups regarding the requirement of neonatal ventilation therapy (any kind), the requirement of kidney replacement therapy (KRT) and the survival over the first year of life using Chi-square test (Table 2).

**Table 2:** Short- and long-term outcome regarding premature birth before 37 + 0 gestational weeks (gw), neonatal ventilation therapy, kidney replacement therapy and survival over one year of **children with unilateral MCDK plus urinary tract anomalies (group 2)**, unilateral MCDK plus extra-renal anomalies* (group 3), unilateral MCDK plus combined anomalies** (group 4) compared to **children with isolated unilateral MCDK (group 1)**. Results in n (%)

Cohort outcome parameter		Group 1	Group 2	*p* value	Group 3	*p* value	Group 4	*p* value
Neonatal ventilation therapy	Yes	7 (15.56%)	0	0.262	6 (50%)	0.012	3 (42.86%)	0.088
	No	38 (84.44%)	7 (100%)		6 (50%)		4 (57.14%)	
Kidney replacement therapy	Yes	0	0	N/A	0	N/A	1 (16.67%)	0.006
	No	44 (100%)	7 (100%)		11 (100%)		5 (83.33%)	
1-year survival	Yes	31 (100%)	6 (100%)	N/A	7 (87.50%)	0.046	3 (50.00%)	< 0.001
	No	0	0		1 (12.50%)		3 (50.00%)	

*: extra-renal anomalies excluding the urinary tract

**: anomaly of the urinary tract AND extra-renal anomaly apart from the urinary tract

Six neonates in group 3 required neonatal ventilation therapy (50%) which was significantly higher compared to group 1 (*p* = 0.012) One newborn in group 4 required KRT (16.67%; *p* = 0.006). Regarding survival, significantly less neonates in group 3 (*p* = 0.046) and group 4 (*p* < 0.001) survived the first year of life compared to group 1.

Four out of 95 children with unilateral MCDK (0.04%) needed nephrectomy of the affected kidney, all four were within group 1 with isolated unilateral MCDK. Kidney involution was documented for only one child (0.01%) that was also part of group 1.

**Fig. 7:**
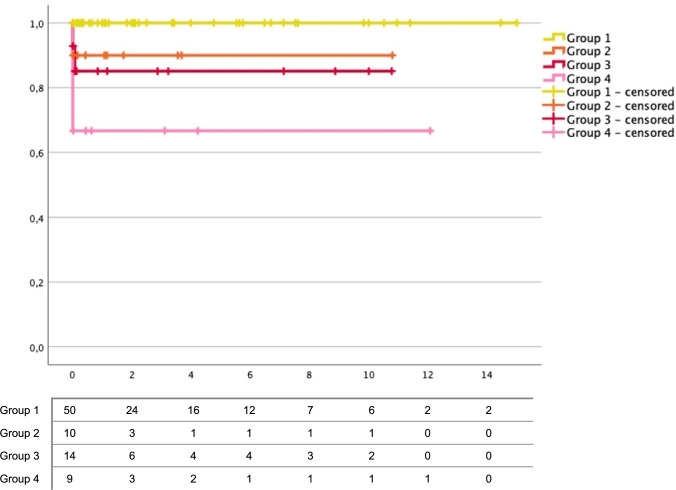
Kaplan–Meier survival analysis illustrating the cumulative survival of **fetuses with isolated unilateral MCDK (group 1)**, **unilateral MCDK plus urinary tract anomalies (group 2)**, unilateral MCDK plus extra-renal anomalies* (group 3) and unilateral MCDK plus combined anomalies** (group 4) (y-axis) in years (x-axis). Neonates with palliative care were excluded. Crosses mark the end of documented survival. Numbers at risk shown in table below. Table below: numbers at risk (n).*: extra-renal anomalies excluding the urinary tract. **: anomaly of the urinary tract AND extra-renal anomaly apart from the urinary tract

A Kaplan–Meier survival analysis was conducted (Fig. 7 (group 1-group 4)). Corresponding numbers at risk can be seen in Table 3. The retrospectively available data that proved survival were assessed. Neonates, that received palliative care, were not included in the survival analysis. Group 1 showed the highest cumulative survival without any reported deaths. Group 2 showed a lower cumulative survival compared to group 1, even lower cumulative survival was calculated within group 3. The lowest cumulative survival was within group 4. The difference regarding the survival between the four groups was proved significant via log-rank test (*p* = 0.002). The Kaplan–Meier curves show that documented deaths occurred only within the first year of life.

**Table 3:** Prognostic relevance of prenatal ultrasound parameters in fetuses with unilateral MCDK on fetal outcome, of which fetuses born prematurely < 37 + 0 gestational weeks in brackets (Chi-square test)

Ultrasound finding outcome parameter		Single umbilical artery		*p* value	Contralateral hydronephrosis		*p* value	Empty urinary bladder		*p* value	Decreasing amount of amniotic fluid throughout pregnancy		*p* value	Anhydramnios		*p* value	Oligohydramnios		*p* value	Pulmonary hypoplasia		*p* value
		Yes	No		Yes	No		Yes	No		Yes	No		Yes	*No*		*Yes*	*No*		*Yes*	*No*	
Neonatal Ventilation Therapy	Yes	5(2)	11(8)	0.011 (0.283)	1(1)	15(9)	0.659 (0.591)	0	16(10)	N/A	4(2)	12(8)	0.154 (0.283)	1(1)	15(9)	0.062 (0.464)	3(3)	13(7)	0.282 (0.171)	1 (1)	15 (9)	0.062 (0.464)
	No	4(0)	51(5)		2(1)	52(4)		0	55(5)		6(0)	49(5)		0	55(5)		5(0)	50(5)		0	55 (5)	
Respiratory adjustment disorder of the newborn	Yes	4(2)	8(6)	0.018 (0.155)	0	12(8)	0.421 (0.104)	0	12(8)	N/A	4(2)	8(6)	0.035 (0.155)	1(1)	11(7)	0.026 (0.333)	2(2)	10(6)	0.516 (0.605)	1 (1)	11 (7)	0.026 (0.333)
	No	5(0)	54(7)		3(2)	55(5)		0	59(7)		6(0)	53(7)		0	59(7)		6(1)	53(6)		0	59 (7)	
Kidney replacement therapy	Yes	0	1(1)	0.733 (0.773)	1(1)	0	< 0.001 (0.011)	0	1(1)	N/A	0	1(1)	0.676 (0.672)	0	1(1)	N/A	1(1)	0	0.006 (0.047)	0	1 (1)	N/A
	No	7(1)	60(12)		2(1)	64(12)		0	67(13)		10(2)	57(11)		0	67(13)		7(2)	60(11)		0	67 (13)	
UTI* requiring antibiotics	Yes	1(0)	4(1)	0.620 (0.685)	1(1)	4(0)	0.075 (0.008)	0	5(1)	N/A	1(0)	4(1)	0.705 (0.685)	0	5(1)	0.780 (0.782)	1(0)	4(1)	0.532 (0.605)	0	5 (1)	0.780 (0.782)
	No	8(2)	57(12)		2(1)	62(13)		0	65(14)		9(2)	56(12)		1(1)	64(13)		7(3)	58(11)		1(1)	64(13)	
1-year survival	Yes	4(1)	43(12)	0.058 (0.101)	3(2)	43(11)	0.792 (0.672)	0	47(13)	< 0.001 (0.008)	6(2)	41(11)	0.590 (0.551)	0	47(13)	< 0.001 (< 0.001)	8(3)	39(10)	0.524 (0.448)	0	47 (13)	< 0.001 (< 0.001)
	No	1(1)	1(1)		0	1(1)		1(1)	1(1)		0(0)	2(2)		2(2)	2(0)		0	2(2)		2 (2)	0	

Results in *n*

*: *UTI* urinary tract infection

### Association of additional ultrasound findings with adverse fetal outcome

Ultrasound parameters observed prenatally were investigated toward their potential association with adverse fetal outcomes using Chi-square test (Table 4). Neonates that received palliative care were excluded from this analysis. Numbers for premature born neonates were calculated in addition. Pulmonary hypoplasia was diagnosed in case of a discrepancy between abdominal and thoracic circumference in favor of the abdominal circumference or reduced measurable lung area which are established methods for the prenatal sonographic diagnosis of pulmonary hypoplasia prenatally [31].

Neonates with the prenatal finding of a SUA revealed higher rates of required neonatal ventilation therapy (55.5%; *p* = 0.011) and respiratory adjustment disorder postnatally (44.44%; *p* = 0.018). There was no fetus with SUA in need of KRT. Newborns with SUA did not have a higher rate of urinary tract infections (UTI). Furthermore, SUA was associated with lower survival rates after one year of life. 80% of the children with SUA survived over one year (*p* = 0.058). The results were similar for premature born neonates, respectively.

Prenatal diagnosis of contralateral hydronephrosis was associated with required neonatal ventilation therapy in 33.33% (*p* = 0.659), with the only child requiring KRT (33.33%; *p* < 0.001) and more UTIs that required antibiotic therapy (33.33%). Fetal hydronephrosis was not associated with a higher rate of respiratory adjustment disorder (*p* = 0.421). The likelihood of surviving over one year was not decreased in the presence of contralateral hydronephrosis also for premature born neonates. Prenatal sighting of an empty urinary bladder was associated with an increased likelihood of not surviving the first year of life (p < 0.001) also for only premature born neonates (*p* = 0.008).

Observing decreasing amounts of amniotic fluid throughout pregnancy was associated with a higher likelihood of neonatal ventilation therapy (*p* = 0.154) and with an increased rate of respiratory adjustment disorders of the newborn (*p* = 0.035), same for prematurely born neonates only. Reduction of amniotic fluid throughout pregnancy was not associated with lower survival rates over one year (*p* = 0.447). Within the cohort of premature newborns, trends were the same but no significant results.

The prenatal finding of anhydramnios was associated with higher rates of neonatal ventilation therapy (*p* = 0.062) and respiratory adjustment disorders of the newborn (*p* = 0.026). Furthermore, anhydramnios was corresponding with lower survival rates over one year in all newborns as well as in premature newborns only (*p* < 0.001).

The sighting of oligohydramnios correlated with higher rates of neonatal ventilation therapy (*p* = 0.282/0.171), respiratory adjustment disorders of the newborn (*p* = 0.516), need for KRT (*p* = 0.006) and UTIs requiring antibiotic therapy (*p* = 0.532). Fetuses with oligohydramnios showed similar survival rates over the first year of life compared to fetuses with normal amount of amniotic fluid (*p* = 0.688). Premature newborns with oligohydramnios showed higher rates of needed ventilation therapy (*p* = 0.171).

Neonates with prenatally diagnosed pulmonary hypoplasia displayed an increased likelihood of neonatal ventilation therapy (*p* = 0.062) and respiratory adjustment disorder (*p* = 0.026) while they showed no higher rate of UTIs requiring antibiotic therapy. The likelihood of death occurring within one year after birth was increased in the presence of pulmonary hypoplasia in all children as well as in prematurely newborns only (*p* < 0.001).

Overall, in our cohorts SUA, empty urinary bladder prenatally, anhydramnios and pulmonary hypoplasia had the biggest impact of fetal survival.

## Discussion

The findings of this study underline the high diagnostic accuracy of prenatal ultrasound identifying MCDK during fetal life with a detection rate of 88.79%. A similar diagnostic accuracy has been reported by other studies [10, 13].

Bilateral MCDK is known to be associated with a poor fetal prognosis due to the near total to total loss of renal function and pulmonary hypoplasia [13, 14, 16]. The cohort of fetuses with bilateral MCDK did not survive beyond the neonatal stage, the longest survival was six days. Additionally, 50% of the cohort of fetuses with bilateral MCDK revealed additional anomalies hinting toward potentially underlying syndromes or genetic variants [32]. Only two of these pregnancies underwent genetic testing; no pathological results were revealed.

As previously indicated in other studies, the reason for not identifying potential genetic diseases could be the limited application of CMA or exome sequencing as mostly chromosome analysis was performed in the past [17, 32]. Unlike during most of the time when the cases analyzed in this study were reported (2005 to 2022), CMA and exome sequencing have now become widely available. Whole genome sequencing only became FDA approved in 2018 after being possible since 2010 [33]. Several CNVs and single nucleotide variants (SNV) are associated with renal malformations [32, 34]. It seems reasonable to assume that with the wide availability of CMA and exome sequencing the vastness of known genetic variants will continue to grow rapidly at the expense of MCDK that is now considered a spontaneous malformation.

The high rate of patients declining genetic diagnostics despite it being offered (27,18%) within our cohort could have cultural reasons, for many becoming parents diagnosing a genetic disease would not have any consequences regarding the pregnancy.

The knowledge of the good prognosis of isolated unilateral MCDK is well established and further underlined by this study [10, 19]. The gender distribution in our cohort of fetuses with unilateral MCDK was 45.26% female, which goes along with other studies [13, 14]. The reason for the higher male incidence has not yet been established in literature. With focus on the pregnancy outcome and long-term outcome, fetuses with unilateral MCDK were distributed into four groups: group 1 (fetuses with isolated unilateral MCDK), group 2 (fetuses with unilateral MCDK plus anomalies of the urinary tract), group 3 (fetuses with MCDK plus extra-renal anomalies apart from the urinary tract) and group 4 (fetuses with MCDK plus anomalies of the urinary tracts and extra-renal anomalies apart from the urinary tract). The analysis could demonstrate significant differences between the four groups regarding the pregnancy outcome as well as the long-term pediatric outcome. The statistical analysis revealed significantly fewer live births in group 2, group 3 and group 4 compared to group 1. IUFD and TOP occurred or was performed significantly more often in group 2; same as TOP and palliative care in group 4 compared to group 1. Prematurity was significantly more common within group 2, group 3 and group 4 compared to group 1. Comparing APGAR scores, group 4 had significantly lower APGAR scores compared to group 1. There were no severe differences in the mean arterial cord blood pH between the groups. The mean arterial cord blood pH was > 7.20 within all groups which is not only understood as nonacidemic but also associated with a low rate of adverse neonatal outcome events [35]. Therefore, an effect or bias of acidemia at birth on our overall outcome parameter analysis can be mostly ruled out. The significantly lower gestational age at birth in group 3 and group 4 is most likely caused due to concerns of fetal distress and non-well-being resulting from a combination of fetal abnormalities [36]. Neonates from group 3 required neonatal ventilation therapy significantly more often and demonstrated a lower probability of surviving during the first year of life. Within our cohort, only one child out of group 4 needed KRT so the informative value in this analysis is very limited. The rate of needed nephrectomies was low, only four out of 95 children (0.04%) with unilateral MCDK, all within group 1. This result goes along with other studies results that reported nephrectomy only being required in special cases [37, 38]. Nevertheless, within our cohort of unilateral MCDK, only one child (0.01%) was reported with involution of the affected kidney, it belonged to group 1. This could be explained not only by the retrospective design of this study but also by the fact that in case of involution of the affected kidney and normal contralateral kidney function, the involution is clinically inapparent. The rate of involution appears to be up to 60% and can be observed within 10–15 years [11, 37, 38].

The results of this study not only support existing literature’s results but moreover imply that a categorization of fetuses with MCDK depending on the existence of additional anomalies is a reasonable approach in prenatal diagnostics and counseling. We assume that the accumulation of IUFD and TOP in group 2 may be caused by severe impairment of the contralateral renal function due to malformations. This could also be underlined by significantly higher serum creatinine levels within group 2 compared to group 1. For example, megaureter is known to be regularly accompanied by kidney dysplasia [39]. High degree hydronephrosis can result in almost complete loss of kidney function. The appearance of MCDK plus further anomalies of the urinary tract should not be seen as a spontaneous appearance but should rather raise suspicion for a potentially underlying genetic variant inhibiting normal embryologic development of the urinary tract. Identifying a genetic etiology provides essential information for counseling of the families regarding both short- and long-term outcomes, including the potential development of end-stage renal disease [1, 3, 5, 32]. Multiple genes are known to control the embryological milestones of kidney development. Variants in the *PAX2* gene have been revealed to lead to CAKUT including MCDK in animal models [40]. A recent study implies that variants of the *PAX2* gene in humans are not involved in the development of MCDK but could prove an association of *PAX2* variants and VUR [41]. Variants in the human *TSHZ3* gene, on the other hand, have not only been proved to cause MCDK but also to be associated with a higher rate of hydronephrosis and hydroureter [42]. The higher rate of TOP, palliative care and APGAR scores in group 4 as well as higher rates of neonatal ventilation therapy, renal replacement therapy and differences regarding the long-term survival picture a generally higher morbidity within group 3 and group 4 compared to group 1. The knowledge that genetic variants do not only impair renal development but also cause various anomalies explains the significantly higher rates of adverse outcome parameters. For example, Meckel–Gruber syndrome being a ciliopathy caused by mutations in numerous genes, the multicystic dysplastic kidneys are only one of many possible phenotypic malformations among encephalocele and polydactyly [27].

It is worth noticing that each pregnancy reported in this study was monitored based on existing guidelines and individual requirements, there were no strict monitoring strategies differing between groups as the groups were only established later within this retrospective analysis. By applying the offered categorization and integrating genetic evaluation into the diagnostic process, clinicians could improve the accuracy of MCDK diagnosis, differentiate from other cystic renal diseases and provide more precise prognostic insights [5]. Establishing a genetic diagnosis not only aids in personalized patient management but also contributes to a better understanding of the underlying mechanisms of congenital kidney anomalies. Moreover, genetic diagnostics can assist in identifying alternative causes of renal dysplasia, further improving clinical management [28].

Kaplan–Meier survival analysis revealed significant differences regarding the survival of children within group 1, group 2, group3 and group 4. This result underlines the study’s core statements. The curves show that deaths were only documented within the first year of life, closely looking at the data mostly within the first days or weeks of life. Firstly, this could be explained by absence or almost absence of kidney function that is life limiting soon after birth. Secondly, due to the retrospective design, children could have presented themselves in another clinic and the data of a potential death or complication are most likely underreported within our data. Thirdly, children with low impact of their kidney malformation might not have the need to see the clinic again. Therefore, retrospective data on long-term survival especially within group 1 are expected to be highly limited in this study.

Ultrasound findings such as SUA (single umbilical artery), anhydramnios and pulmonary hypoplasia were associated with adverse outcome parameters, as shown by others [11, 12, 21]. In recent studies, isolated SUA has been proved not to be linked with a poor fetal outcome, contrarily to SUA in combination with other fetal malformations. Therefore, in the presence of SUA, thorough search for other anomalies is strongly recommended [43]. When diagnosing SUA combined with additional findings, genetic testing is recommended due to a higher rate of chromosomal aberrations [44]. The latter supports this analysis’ results. In our cohorts, SUA in combination with MCDK was associated with a higher rate of adverse events. This association raises the possibility that non-renal etiologies (e.g., syndromic or vascular anomalies) may be the actual driver of poor prognosis. But because of the few genetic results in this cohorts, this point remains unclear. The results support the assumption that anhydramnios is a sonographic sign of renal impairment if premature rupture of the membranes is ruled out [21]. Fetal pulmonary hypoplasia is commonly caused by the absence of amniotic fluid that affects the pulmonary development [45]. Additionally, a significant correlation with a higher rate of neonatal respiratory adjustment disorders and the reduction of amniotic fluid was detected. A possible explanation can be progredient loss of renal function throughout pregnancy if the contralateral kidney function is impaired. No clear association with unfavorable fetal outcome parameters could be seen in case of oligohydramnios. This could be explained by the fact that oligohydramnios can be a sign of placental insufficiency [46]. By including the numbers and p-values for prematurely born neonates, prematurity could be ruled out as a bias for KRT, UTI requiring antibiotics and one-year survival. Nevertheless, p-values were strongly affected by the prematurity when assessing the rates of respiratory adjustment disorder. Therefore, regarding this outcome, a bias of our analysis caused by prematurity is to be assumed. Further parameters including anomalies of the central nervous system, cardiologic anomalies and anomalies of the genital tract were assessed. Due to the small case numbers, there were no significant results.

Limitations of the available data confined this retrospective study. The relatively small amount of long-term data prohibited the appliance of a logistic regression model. In the future, prospective studies, preferably as a registry, should further investigate the correlation between prenatal findings and outcome parameters in fetuses with MCDK.

The proposed classification system for unilateral MCDK is practical and based on meaningful clinical observations. It is not a dramatic departure from existing frameworks, but it does offer a structured way to think about risk stratification. That said, it is based on a single-center cohort, and validation in other settings would be needed before it could be widely adopted. The representivity of our cohort might be impaired by the fact that our clinic offers multidisciplinary care for maternal, neonatal and nephrological diseases. Therefore, as a center for high-risk patients, our cohort might also be biased toward high-risk or more severe cases. In literature, the rate of associated malformations in the presence of MCDK is lower than in our cohort [10].

The strength of this study is that it analyzes one of the largest single-center cohorts with prenatally diagnosed MCDK-patients to be found in the currently existing literature.

## Conclusion

MCDK is a common prenatal diagnosis. Bilateral MCDK is associated with an unfavorable prognosis, whereas isolated unilateral MCDK is associated with an overall good fetal and neonatal outcome. This study was able to demonstrate that, in presence of unilateral MCDK, additional findings such as SUA, contralateral hydronephrosis, empty urinary bladder, anhydramnios and pulmonary hypoplasia can indicate a higher risk for an adverse fetal and neonatal outcome. It is to be expected that other additional extra-renal anomalies in the presence of MCDK like malformations of the central nervous system, the cardiovascular system or the genital tract are also associated with an unfavorable fetal outcome. Therefore, the presence of MCDK with additional malformations implies neonatal as well as genetic counseling and further genetic diagnostics including chromosome analysis, CMA and exome sequencing should be offered. A categorization of MCDK-fetuses depending on the presence of additional anomalies is a reasonable approach as the fetal outcomes could be demonstrated to differ significantly.

## Data Availability

The data that support the findings of this study are available from the corresponding author upon request.
